# Improving the Accuracy of Rainfall Prediction Using Bias-Corrected NMME Outputs: A Case Study of Surabaya City, Indonesia

**DOI:** 10.1155/2022/9779829

**Published:** 2022-04-27

**Authors:** Defi Y. Faidah, Heri Kuswanto, Sutikno Sutikno

**Affiliations:** ^1^Department of Statistics, Institut Teknologi Sepuluh Nopember, Surabaya 60111, Indonesia; ^2^Department of Statistics, Universitas Padjajaran, Bandung 45363, Indonesia; ^3^Center for Disaster Mitigation and Climate Change, Institut Teknologi Sepuluh Nopember, Surabaya 60111, Indonesia

## Abstract

Generating an accurate rainfall prediction is a challenging work due to the complexity of the climate system. Numerous efforts have been conducted to generate reliable prediction such as through ensemble forecasts, the North Multi-Model Ensemble (NMME). The performance of NMME globally has been investigated in many studies. However, its performance in a specific location has not been much validated. This paper investigates the performance of NMME to forecast rainfall in Surabaya, Indonesia. Our study showed that the rainfall prediction from NMME tends to be underdispersive, which thus requires a bias correction. We proposed a new bias correction method based on gamma regression to model the asymmetric pattern of rainfall distribution and further compared the results with the average ratio method and linear regression. This study showed that the NMME performance can be improved significantly after bias correction using the gamma regression method. This can be seen from the smaller RMSE and MAE values, as well as higher *R*^2^ values compared with the results from linear regression and average ratio methods. Gamma regression improved the *R*^2^ value by about 30% higher than raw data, and it is about 20% higher than the linear regression approach. This research showed that NMME can be used to improve the accuracy of rainfall forecast in Surabaya.

## 1. Introduction

Rainfall is one of the most important climate variables that impacts various sectors of life. Indonesia has a diverse characteristic of rainfall, indicated by three general rainfall patterns: monsoonal, equatorial, and local rainfall patterns [1,2]. An accurate rainfall prediction is required to mitigate the negative impacts of extreme rainfall events. One of the efforts to generate an accurate rainfall prediction is through the ensemble method, which is intended to capture the uncertainty induced by several factors. To deal with this, various scenarios are needed to simulate future weather climate projection by involving large-scale data, as has been conducted by many climate centres in developed countries. Data availability also becomes an important issue in building a good rainfall prediction model [3].

The NMME is one of the ensembles forecast products generated from the experimental multimodel seasonal forecasting system consisting of coupled models from North American modelling centres. It has been developed to produce more accurate rainfall prediction either in short, medium, or long term [4]. The NMME outputs have been widely used in previous studies such as evaluation of the predictive performance of rainfall [5–7] and surface temperature [8], prediction of monthly sea surface temperature as a form of the ENSO index [9], prediction of seasonal rainfall [10,11], the onset of seasonal droughts globally [12], and long-term climate predictions [13]. NMME data have been proven to be a reliable tool for various monitoring systems globally. However, the validation of the performance in any specific location has not been much explored. This paper investigates the performance of NMME to be used for forecasting rainfall in Surabaya city, Indonesia.

The NMME data tend to be biased towards the observational data. Therefore, a postprocessing technique with bias correction is needed to improve the NMME performance. There have been many bias correction methods developed, e.g., average ratio method and regression-based methods. Lenderink et al. [14] used the average monthly observed rainfall ratio with the data to be corrected. The method has been applied also in [15] to correct the bias of the satellite precipitation product. Multiple linear regression is one of the simple methods that can be used for bias correction [16]. Multiple linear regression assumes that rainfall has a normal distribution pattern. However, it has been proven in many studies that rainfall has a nonnormal distribution due to the presence of extreme observations. Sloughter et al. [17] argued that normal distribution cannot be used to describe the rainfall patterns. Therefore, bias correction methods developed without considering this fact might lead to some degrees of bias, which thus influences the forecasting performance.

The presence of extreme observations in rainfall data leads to an asymmetrical distribution with a long tail on the right, which can be approximated by using gamma distribution. This paper proposes a bias correction method using gamma regression and further evaluates the performance of gamma regression in correcting the bias of NMME data compared to the average ratio method and linear regression. Furthermore, we will evaluate the performance of each ensemble member on forecasting rainfall in Surabaya city, Indonesia. The results of this research can be used as a reference to develop a weather forecast system using NMME products with higher accuracy.

## 2. Materials and Methods

### 2.1. Study Area and Data

**Figure 1:**
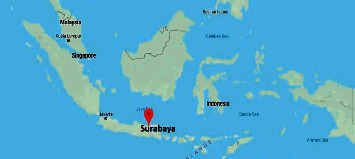
Map of Indonesia and spatial location of Surabaya city.

This research focuses on rainfall forecasting in Surabaya city, Indonesia, which is also the largest city and the centre of the provincial government in East Java. The spatial location of Surabaya city can be seen in Figure 1.

In general, Surabaya has a tropical climate characterized by two seasons, namely, dry and rainy seasons. Surabaya also plays a vital role in the hydrological field in East Java due to its strategic location. Moreover, extreme rainfall happened during rainy season can easily lead to flood in the city. To minimise the impact of flood events, proper planning and countermeasures are needed. It includes good mitigation strategies through the availability of accurate information about rainfall prediction.

The data used in this study comprise monthly prediction of precipitation generated from the ensemble models (NMME) which can be accessed from the NMME website at https://www.cpc.ncep.noaa.gov/products/NMME/ and daily rainfall observation data collected from Juanda Meteorological Station (located in Surabaya city) spanning from January 1982 to December 2018. In this study, the NMME models used for precipitation prediction are generated from the Canadian Coupled Climate Model version 3 (CanCM3), Canadian Coupled Climate Model version 4 (CanCM4), Community Climate System Model version 3 (CCSM3), Community Climate System Model version 4 (CCSM4), and Geophysical Fluid Dynamics Laboratory Climate Model version 2p1 (GFDL-CM2p1). The analysis in this paper is limited to 1-month lead forecasts.

### 2.2. Bias Correction Models

This study uses gamma regression as one of the proposed methods for bias correction of the NMME data. The bias correction resulted from gamma regression was compared with other methods, namely, the average ratio method and linear regression. Furthermore, we evaluate the performance (accuracy) of the prediction by comparing NMME rainfall data before and after the correction. Before bias correction hereafter is denoted as raw data. The main feature of bias correction is the determination of the correction factor. This study proposes using gamma regression to obtain the correction factor, which is thus compared with the average ratio method and linear regression.

### 2.3. Average Ratio Method

Bias correction using the average ratio method [18] was carried out by using the ratio of the average monthly rainfall of the observation data ${\bar{P}}_{\text{obs}}\left(t\right)$ to the average monthly rainfall of the NMME data $\left({\bar{P}}_{\text{model}}\left(t\right)\right)$ for each model according to equation (1).

(1) $$\begin{array}{c} P_{\text{cor}}\left(t\right)=P_{\text{model}}\left(t\right)\times \frac{{\bar{P}}_{\text{obs}}\left(t\right)}{{\bar{P}}_{\text{model}}\left(t\right)}, \end{array}$$

where *P*_obs_ is rainfall observation and *P*_model_ is NMME rainfall forecast.

### 2.4. Simple Linear Regression Model

The regression-based method is one of the popular bias correction methods and has been proven to be able to correct bias well. Linear regression is a popular way of analysing data described in linear models in which one variable is considered as the explanatory variable and the other as the dependent variable [19]. The model is obtained by finding a fit through the points that form a linear line. The line connects the explanatory and dependent variables. The goal of this model is to find the parameters which minimise the square of the error. Regression models are defined according to [19–21]

(2) $$\begin{array}{c} y=x^{T}\beta , \end{array}$$

where *y* is the response variable and *x* is the predictor variable. The first step in using the regression model in bias correction is to determine the bias correction factor. The bias correction factor is obtained from the regression coefficient *β*. In this method, the coefficient is estimated by minimizing the residual or prediction error. The smaller the error value, the better the regression equation model.

### 2.5. Gamma Regression

Gamma regression describes the relationship between response variables with gamma distribution and predictor variables [22,23]. Gamma distribution is a type of continuous probability distribution that can be applied in various fields, especially when the data distribution is positively skewed. According to the requirements of the gamma distribution, the response variable is continuous data with nonnegative value and has a constant coefficient of variation [24–26]. Furthermore, for extreme analysis, this method is more appropriate because it considers the distribution of rainfall, by assuming that its observation and NMME data follow gamma distribution with the probability density function defined as follows [27]:

(3) $$\begin{array}{c} f\left(y;\upsilon ,\gamma \right)=\frac{1}{\gamma^{\upsilon }\Gamma \left(\upsilon \right)}y^{\left(\upsilon -1\right)}e^{\left(\left(-y\right)/\gamma \right)},\;y>0,\\ f\left(y;\upsilon ,\gamma \right)=0,\;y\leq 0, \end{array}$$

where *y* is monthly rainfall, *υ* and *γ* are the shape and scale parameters, respectively, and Γ(*υ*) is a Gamma function, solved by a factorial function Γ(*υ*)=(*υ* − 1)!. Based on equation (3), the gamma regression model can be formed which is denoted by *G*(*μ*, *φ*), where *μ*=*E*(*y*) is mean function and *φ* > 0 is the dispersion parameter. If the parameters *υ* and *γ* are reparameterized to *υ*=*ϕ*^−1^ and *γ*=*μφ*, then equation (3) is defined to be [28].

(4) $$\begin{array}{c} f\left(y;\mu ,\varphi \right)=\frac{1}{\Gamma \left(\varphi^{-1}\right)}{\left(\frac{1}{\mu \varphi }\right)}^{\varphi^{-1}}y^{\varphi^{-1}-1}e^{\left(\left(-y\right)/\mu \varphi \right)},\;y>0. \end{array}$$

The gamma distribution is a special form of the exponential family that follows equation (5) as follows:

(5) $$\begin{array}{c} f\left(y;\theta ,\varphi \right)=\exp\left[\frac{y\theta -b\left(\theta \right)}{a\left(\varphi \right)}+c\left(y,\varphi \right)\right], \end{array}$$

where *θ* indicates the location parameter, *φ* is the dispersion parameter, and *b*(*θ*) is the cumulant function. Equation (5) can be rewritten by changing the parameter *θ*=1/*μ*, *a*(*φ*)=*φ*, and *b*(*θ*)=−ln(*μ*) so that equation (6) is obtained.

(6) $$\begin{array}{c} f\left(y;\theta ,\varphi \right)=\exp\left[\frac{y/\mu -\left(-\ln\left(\mu \right)\right)}{-\varphi }+\frac{1-\varphi }{\varphi }\ln\left(y\right)-\frac{\ln\left(\varphi \right)}{\varphi }-\ln\;\Gamma \left(\frac{1}{\varphi }\right)\right]. \end{array}$$

Gamma regression models are often modelled using a reciprocal link function which is expressed as a linear combination of covariate variables *g*(*μ*_*i*_)=1/*μ*_*i*_=**x**_*i*_^*T*^*β* where **x**_*i*_^*T*^=(*x*_*i*1_, ⋯,*x*_*ip*_)^*T*^. The most used method for estimating the gamma regression parameter is maximum likelihood estimation. Based on equation (6), the likelihood function is formed, and we can obtain

(7) $$\begin{array}{c} L=\log\;l\left(y;\theta ,\varphi \right)=\sum_{i=1}^{N}\left[\frac{y_{i}x_{i}^{T}\beta -\log\left(x_{i}^{T}\beta \right)}{1/\varphi }+c\left(y_{i},\varphi \right)\right], \end{array}$$

where *c*(*y*_*i*_, *φ*)=((1 − *φ*)/*φ*)ln(*y*) − ln(*φ*)/*φ* − ln  Γ(1/*φ*).

To get the maximum likelihood estimator, equation (7) is derived against the estimated parameters and equated to zero. The result of the first derivative cannot be solved analytically, and hence, it is solved by using a numerical iteration method.

### 2.6. Performance Evaluation

After correcting the NMME model, the model accuracy needs to be assessed. Phogat et al. [18] stated that the evaluation of bias correction results is particularly important to determine the performance of the NMME model before and after correction. In this study, the models' accuracy is assessed with *R* squared (*R*^2^), root mean square error (RMSE), and mean absolute error (MAE).

#### 2.6.1. *R*-Square (*R*^2^)

The R-square or coefficient of determination is a simple measure and is often used to determine the performance of the regression model. The R-square value provides an overview of the suitability of the independent variable in predicting the dependent variable. The calculation of the R-square is as follows [29]:

(8) $$\begin{array}{c} R^{2}=\frac{\sum_{i=1}^{N}{\left(y_{i}-{\hat{y}}_{i}\right)}^{2}}{\sum_{i=1}^{N}{\left(y_{i}-\bar{y}\right)}^{2}}\times 100\%, \end{array}$$

where *y*_*i*_ is the observation value in period *i*, ${\hat{y}}_{i}$ is the forecast value in period *i*, and *N* is the number of observations.

#### 2.6.2. Root Mean Squared Error (RMSE)

The RMSE is a popular measure of evaluating the goodness of forecasting models. RMSE is the root value of the mean squared forecasting bias. The forecasting bias can be interpreted as the difference between the predicted value and the actual observed value. The RMSE formula can be expressed as follows [30]:

(9) $$\begin{array}{c} \text{RMSE}=\sqrt{\frac{\sum_{i=1}^{N}{\left(y_{i}-{\hat{y}}_{i}\right)}^{2}}{N}}, \end{array}$$

where each component of RMSE is defined as in equation (8).

#### 2.6.3. Mean Absolute Error (MAE)

Mean absolute error (MAE) is the absolute average of the prediction errors, regardless of the positive or negative sign. The use of MAE in the evaluation of forecasting results can see the level of accuracy. The equation for calculating the mean absolute error (MAE) is as follows [30]:

(10) $$\begin{array}{c} \text{MAE}=\frac{1}{N}\sum_{i=1}^{N}\left|y_{i}-{\hat{y}}_{i}\right|. \end{array}$$

## 3. Results and Discussion

### 3.1. Results

Substantial constraints and problems that are commonly encountered in conducting rainfall analysis are the limited availability and low quality of observation rainfall data due discontinuous recording, lots of missing data, and other factors [4]. Generating rainfall prediction by using ensemble outputs is one of the solutions to solve the problems. However, the performance of the ensemble forecast should be validated, and, in some cases, they will need to be postprocessed or calibrated.

**Figure 2:**
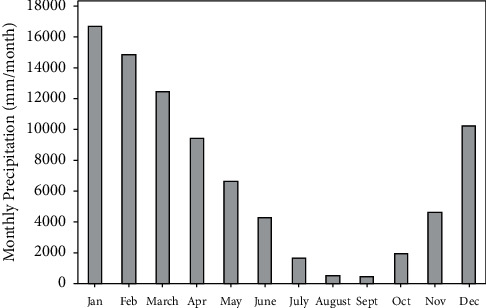
Monthly rainfall pattern observed from the Juanda Meteorological Station.

**Figure 3:**
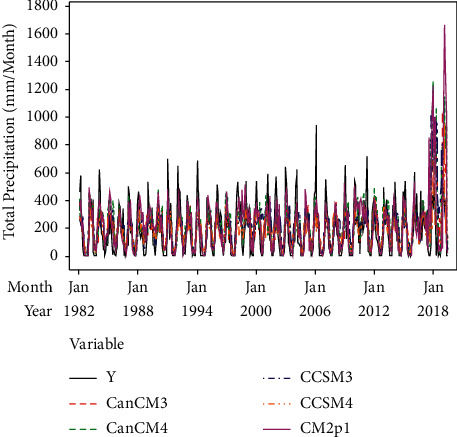
Time series plot between ensemble prediction and rainfall observation.

**Figure 4:**
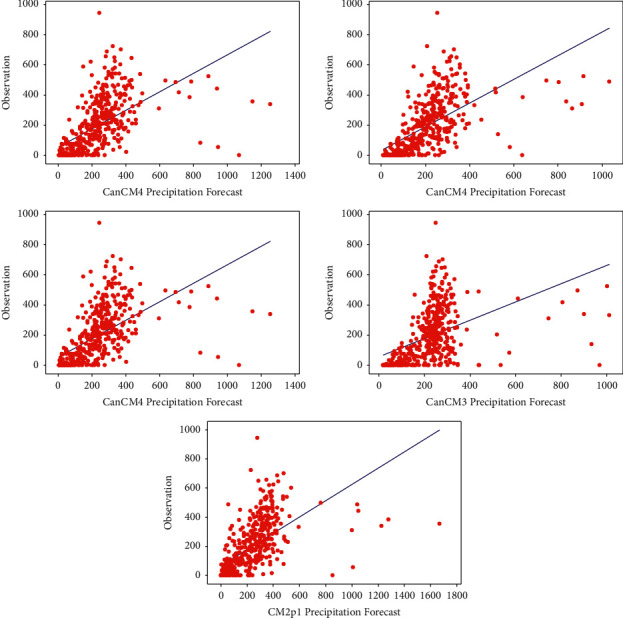
Scatterplots of monthly rainfall observation against NMME forecast.

Figure 2 shows that the rainfall pattern in Surabaya forms the letter U, which is the common characteristic of the monsoon rainfall pattern. The peak of the rainy season occurs in January, while the peak of the dry season occurs in August. The time series plots of the forecast results generated by the five ensemble members and the observation of rainfall data at Juanda Station are shown in Figure 3. The time series plot is used to determine the relationship between each ensemble member's forecast value with the observation data. Moreover, time series plots can show the extent to which the forecast results can capture rainfall patterns well. Based on Figure 3, the rainfall forecast generated from each ensemble member deviates from the observation, with the pattern that resembles the observation well such as the seasonality pattern. The deviation between observation and forecast indicates a bias. In addition to using time series plots, initial identification of the ensemble's forecast can be shown through scatterplots as can be depicted in Figure 4.

Figure 4 shows the relationship between observed rainfall and the NMME forecast for each model. In general, all models have similar patterns. The points above the line indicate that the forecast values are lower than the observation or it is said to be underestimated. Meanwhile, the points below the line indicate that the forecast values are higher than the observations, or they are overestimated. The preliminary identification results show that the ensemble outputs are biased towards the observation indicated by overestimated and underestimated forecasts. Hence, it is necessary to correct the bias, where in this paper, it will be conducted by using the average correction method, linear regression, and gamma regression. After correcting the bias, the performance of each ensemble forecast is evaluated by using RMSE, MAE, and *R*^2^ as listed in Table 1.

Based on the values in Table 1, the comparison between observation and the NMME forecasts for each ensemble model shows different performance. Table 1 shows that the forecast values corrected using gamma regression have the lowest RMSE and MAE values for each ensemble member. Moreover, bias correction with gamma regression resulted in higher *R*^2^ values compared to two other methods. We can also see that there is a substantial improvement on the bias reduction between after bias correction using gamma regression. The average ratio method removes the bias slightly, while two regression methods remove the bias significantly proven by significant reduction of RMSE and MAE, and significant improvement of the *R*^2^ value. Nevertheless, the gamma regression outperforms the linear regression.

**Figure 5:**
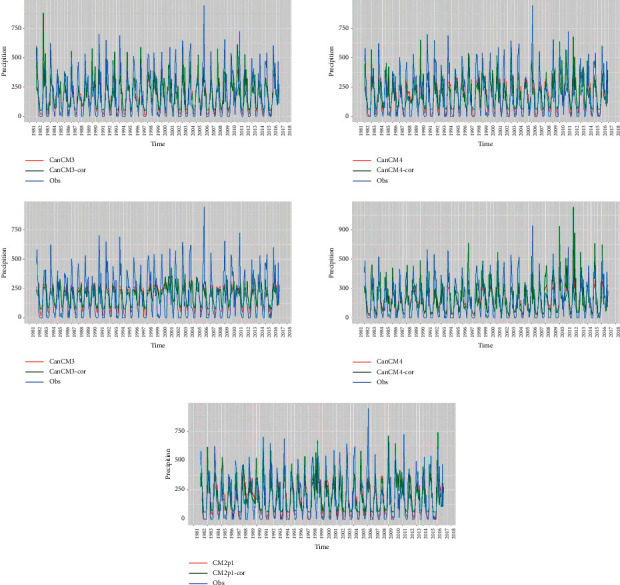
Comparison of the observed rainfall patterns, NMME dataset before and after correction, for each ensemble member.

Figure 5 shows the time series plots of the observed rainfall pattern, NMME rainfall forecast, and NMME corrected by gamma regression for each ensemble model. Based on Figure 5, we observed that the bias correction using gamma regression shifted the NMME forecast to the level closer to the observed rainfall data. We also observed a consistent pattern between the corrected NMME data and the observed rainfall. This indicates that the bias correction is able to preserve the seasonal pattern in the data, and therefore, the bias-corrected NMME rainfall data can be used to represent the observed rainfall data. Model CM2p1 shows the best performance compared to other models. This can be seen from the corrected rainfall forecast, which has a value close to the observation. In addition, the CM2p1 model has the highest *R*^2^ value and the smallest RMSE value. On the other hand, the CCSM3 model shows the worst performance compared to the other models.

### 3.2. Discussion

NMME forecast generates ensemble forecasts from different models to capture the uncertainty [6,31]. The forecasts were generated from numerical weather prediction involving complex computational systems. The NMME bias needs to be corrected to produce valid and reliable rainfall forecasts [29–33]. The simplest method of bias correction is the average ratio. The average ratio method's basic principle is to compare the average value of the observed rainfall data and the average forecast results of the NMME model. Besides the simple calculation process, the average ratio method has weaknesses. Hossain [34] reported that mean values tend to be influenced by extreme values in the data. The extreme rain phenomenon that often occurs results in a series of rainfall observation data containing many extreme values. Therefore, the results of the average ratio method correction cannot represent the observed data. Based on Table 1, it can be seen that RMSE value from the average ratio method of the corrected data differs slightly with the uncorrected data.

Ninyerola et al. [16] used linear regression as a bias correction method and proved that the method is a simple technique to predict rainfall with adequate good performance. Linear regression analysis is a statistical method used to explain the effect of two or more independent variables on the dependent variable. Rainfall observation is used as the dependent variable, while NMME data is used as the independent variable. Based on the results of the bias correction in Table 1, it can be seen that the RMSE value after bias correction has decreased significantly compared to RMSE of the uncorrected data. However, even though it is decreased, the RMSE value resulted from the bias correction of the average ratio method, and multiple linear regression was not significantly different. This is because the principle of multiple linear regression assumes that the data follow a normal distribution, which is not really the case of rainfall distribution. The rainfall pattern does not have a symmetrical distribution pattern but is skewed on one side due to the influence of extreme precipitation.

Gamma regression proposed in this paper is an alternative to the bias correction method for rainfall data. The use of gamma regression is adjusted to the rainfall distribution pattern, which tends to be skewed. The skewed gamma distribution pattern can accommodate the observed values of extreme rainfall. This study found that the RMSE and MAE values are significantly lower compared to the error of the average ratio and linear regression methods. This means that the bias correction using gamma regression is better than the average ratio and linear regression methods. Moreover, the *R*^2^ values after correction with the Gamma regression showed the highest compared to the others.

In a climate change impact study, using an ensemble model has been proven to be capable of improving rainfall forecasts compared to a single model [35–37]. However, too many models used will make the forecast results ineffective and inefficient. Therefore, each NMME model's performance analysis is used as a reference in selecting several optimal ensemble models to get an accurate ensemble mean [38].

Based on the results in Table 1, the CM2p1 model shows the best performance compared to other models. This can be seen from the corrected rainfall forecast, which has a value close to the observation. In addition, the CM2p1 model has the highest *R*^2^ value and the smallest RMSE value. The CM2p1 model was developed based on data assimilation between the previous GFDL forecasting model and the real-time observation values from several other meteorological institutions. This forecasting model's assimilation results are proven to produce stable and realistic forecasts over a long period, regardless of the large horizontal grid resolution used [39]. The GFDL is an atmospheric model developed by NOAA. This model was developed to facilitate the detection and prediction of climate variability in seasonal to multidecade periods [40].

On the other hand, the CCSM3 model shows the worst performance compared to the other models. The CCSM3 model is designed to produce realistic forecasting of atmospheric patterns with long-lasting and straightforward simulations [41]. However, the CCSM3 model still lacks the quality of forecasts in the tropics. Developing the CCSM4 model produces more realistic forecasting results, especially forecasting atmosphere patterns in the tropics [42, 43].

## 4. Conclusions

This research showed that the NMME rainfall forecasts have a similar pattern as the observed rainfall with some degrees of bias. The NMME data tend to overestimate or underestimate which thus requires bias correction. Based on the values of RMSE, MAE, and *R*^2^, bias correction using gamma regression outperforms the average bias correction and linear regression. Gamma regression can reduce the bias in NMME data significantly and be able to improve the accuracy of the rainfall prediction. The CM2p1 model shows the best performance compared to other models. On the other hand, the CCSM3 model shows the worst performance compared to the other models.

All results presented in this paper are based on analysing NMME data which was drawn from a single point of latitude and altitude. To improve the performance, further study needs to be done by considering the NMME data from several grid points and postprocessing it using some statistical methods such as principle component analysis (PCA). Furthermore, this paper used single iteration to compare the methods. More advanced approach using cross validation or Monte Carlo simulation can be applied for future study, as described by Bokde et al. [44].

## Figures and Tables

**Table 1:** Evaluation of NMME data before and after bias correction.

Evaluation		Model				
		CanCM3	CanCM4	CCSM3	CCSM4	CM2p1
Before correction	RMSE	140.9	151.4	71.1	137.9	144.9
	MAE	104.1	107.6	122.4	101.2	103.9
	R^2^	31.6	30.6	19.7	40.4	34.8

Average ratio	RMSE	140.8	146.1	159.9	136.3	143.3
	MAE	103.3	106.2	123.8	97.1	103.8
	R^2^	38.4	33.7	20.6	42.3	36.2

Linear regression	RMSE	137.3	142.6	158.9	135.5	135.3
	MAE	97.0	96.7	117.7	101.1	90.7
	R^2^	41.6	36.6	29.7	46.2	44.9

Gamma regression	RMSE	133.7	135.9	154.5	132.6	127.6
	MAE	89.8	89.8	85.6	57.6	72.1
	R^2^	67.1	65.6	51.3	67.6	70.4

## Data Availability

The link of the data used to support the findings of this study is included within the article.
